# Effects of *Phytophthora* Inoculations on Photosynthetic Behaviour and Induced Defence Responses of Plant Volatiles in Field-Grown Hybrid Poplar Tolerant to Bark Canker Disease

**DOI:** 10.3390/jof7110969

**Published:** 2021-11-15

**Authors:** Jaroslav Ďurkovič, Tatiana Bubeníková, Adriána Gužmerová, Peter Fleischer, Daniel Kurjak, Ingrid Čaňová, Ivan Lukáčik, Miloň Dvořák, Ivan Milenković

**Affiliations:** 1 Faculty of Forestry, Technical University in Zvolen, T.G. Masaryka 24, 96001 Zvolen, Slovakia; adriana.guzmerova@gmail.com (A.G.); p.fleischerjr@gmail.com (P.F.); kurjak@tuzvo.sk (D.K.); ingrid.canova1@gmail.com (I.Č.); ivan.lukacik@tuzvo.sk (I.L.); 2 Faculty of Wood Sciences and Technology, Technical University in Zvolen, T.G. Masaryka 24, 96001 Zvolen, Slovakia; tatiana.bubenikova@tuzvo.sk; 3 Faculty of Forestry and Wood Technology, Mendel University in Brno, Zemědělská 3, 61300 Brno, Czech Republic; milon.dvorak@seznam.cz; 4 Phytophthora Research Centre, Faculty of Forestry and Wood Technology, Mendel University in Brno, 61300 Brno, Czech Republic; ivan.milenkovic@sfb.bg.ac.rs; 5 The Chair of Forest Protection, Faculty of Forestry, University of Belgrade, Kneza Višeslava 1, 11030 Belgrade, Serbia

**Keywords:** α-cubebene, bark canker, β-caryophyllene, gas exchange, germacrene D, transpiration

## Abstract

Bark cankers accompanied by symptoms of decline and dieback are the result of a destructive disease caused by *Phytophthora* infections in woody plants. Pathogenicity, gas exchange, chlorophyll *a* fluorescence, and volatile responses to *P. cactorum* and *P. plurivora* inoculations were studied in field-grown 10-year-old hybrid poplar plants. The most stressful effects of *P. cactorum* on photosynthetic behaviour were found at days 30 and 38 post-inoculation (p.-i.), whereas major disturbances induced by *P. plurivora* were identified at day 30 p.-i. and also belatedly at day 52 p.-i. The spectrum of volatile organic compounds emitted at day 98 p.-i. was richer than that at day 9 p.-i, and the emissions of both sesquiterpenes α-cubebene and germacrene D were induced solely by the *Phytophthora* inoculations. Significant positive relationships were found between both the axial and the tangential development of bark cankers and the emissions of α-cubebene and β-caryophyllene, respectively. These results show that both α-cubebene and germacrene D are signal molecules for the suppression of *Phytophthora* hyphae spread from necrotic sites of the bark to healthy living tissues. Four years following inoculations, for the majority of the inoculated plants, the callus tissue had already closed over the bark cankers.

## 1. Introduction

*Phytophthora* species are pathogenic fungi-like organisms, belonging to the kingdom Chromista/Stramenopiles and the “SAR” (Stramenopiles, Alveolata, and Rhizaria) supergroup [1]. This genus is among the most destructive plant pathogens of various hosts in agriculture, forestry, and ornamental plantings worldwide [2,3]. The main reasons for their widespread distribution are increased global trade and the introduction of *Phytophthora* pathogens into non-native environments [4,5]. Following their introduction to other continents, they can become invasive and threatening to nonadapted flora which, due to a lack of co-evolution, contain a high number of susceptible species [4]. In European ecosystems, pathogens from the genus *Phytophthora* are causing significant damage to various forest and ornamental woody plants [3]. Examples of the most notorious invasive *Phytophthora* pathogens include *P. cinnamomi* Rands, *P. lateralis* Tucker and Milbrath, *P. plurivora* Jung and Burgess, and *P. ramorum* Werres et al.—all of which cause devastating epidemics to entire forest ecosystems in Europe, America, and Australia [6,7,8,9].

Forestry and ornamental nurseries are recognised as one of the main sources for spreading *Phytophthora* pathogens into natural and seminatural ecosystems [10,11]. This is particularly important in the context of intense establishment of planted forests. Plantations of various poplar (*Populus* spp.) clones are one of the most widespread and most important in terms of easy cultivation, high biomass within a short period, wood quality for different uses, and also for carbon sequestration [12,13]. Since poplar plantations are usually established on floodplains or in wet areas, there is a high risk posed by *Phytophthora* pathogens [14].

There are only a few records of *Phytophthora* pathogens affecting poplar trees, and these hosts were considered to be tolerant to some species of *Phytophthora* [6]. From symptomatic tissue and from rhizosphere soil of white poplar, *P. cactorum* (Lebert and Cohn) Schröter was isolated in the Czech Republic [15] and in Hungary [10], respectively. In addition, during the studies of declines among poplar clones in Croatia, *P*. × *cambivora* (Petri) Buisman and *P. gonapodyides* (H.E. Petersen) Buisman were both isolated from beneath the symptomatic trees [16] and connected to the recorded decline. Further, in a study of the mycological complex present on poplars in Serbia [17], *P. cactorum* and *P. plurivora* were isolated from the rhizosphere soil under poplar trees. Recently, a community of six species was detected in symptomatic and healthy poplar plantations in Serbia, and the aggressiveness of these isolated species was demonstrated following tests for both soil infestation and stem inoculation pathogenicity [14].

Most biotic stress factors have a direct or indirect impact on CO_2_ assimilation, and biotic agents often restrict physiological processes similarly to abiotic factors. For instance, a disturbance to phloem transport may heavily affect the root system [18] and damage to the root system or to xylem tissues may lead to insufficient water supply, stomatal closure, and eventually to hydraulic failure [19,20]. Limited water transport may induce overheating in the leaves as transpiration-mediated cooling is limited, leading eventually to the damage of photosystems II [21]. Host trees often create smaller leaves, altering the synthesis and effectivity of assimilatory pigments [22,23]. Of course, plants have developed a set of defensive mechanisms to survive and prevent losing vitality as the products of photosynthesis are indispensable for the synthesis of carbon-based defence-related compounds [24]. In general, biotic stress factors may amplify and be amplified by abiotic stress [25].

End-products of plant metabolism, such as volatile organic compounds (VOCs), are important molecules released from plant tissues during abiotic or biotic stresses. High-temperature periods in the growing season, mechanical wounding, insect feeding, or fungal attack all induce emissions of distinct VOCs differing in their composition and concentration depending on the given stressful stimulus [26,27,28]. In addition, community members of plants, insects, or fungi interact in response to the released VOCs. Active emissions of VOCs play important roles in priming defence responses, repelling herbivores, attracting their predators, or beneficial microbes for plant immunity [29,30,31,32]. Some terpenoids may act as endogenous defence signals or display antimicrobial, antifungal, and insecticidal activities [33].

In this study, we focussed on the assessment of disease symptoms and induced volatile responses during the 99 day period of measurements in field-grown two-way hybrid poplar following artificial inoculations with *P. cactorum* and *P. plurivora*, which represent two aggressive generalists and are the most common species in field surveys of poplar plantations. Large field-grown trees were chosen as they offer a beneficial advantage over the greenhouse-grown seedlings in the development of more realistic levels of a disease. The objectives for this study were: (i) to identify temporal changes in gas exchange and chlorophyll *a* fluorescence in response to the inoculations and to determine the period when the *Phytophthora* hyphae show the most harmful effects on photosynthetic traits; and (ii) to identify the emissions of volatile organic compounds (VOCs) that are induced by the *Phytophthora* isolates and which may contribute to plant defence responses.

## 2. Materials and Methods

### 2.1. Plant and Oomycete Materials, Study Site, and Measurement Days

Experiments were conducted on 10-year-old clonally micropropagated plants of the hybrid poplar clone T-14, *Populus tremula* L. 70 × (*Populus* × *canescens* (Ait.) Sm. 23), growing in the experimental field plot at Zvolen, Slovakia (48°35′N, 19°08′E, 297 m a.s.l.). Recently, T-14 plants micropropagated in tissue culture showed a better performance for leaf growth traits, dissipation energy of midrib xylem conduit cell walls, and cellulose content than the plants propagated conventionally from root cuttings [34,35]. Artificial inoculations of *P. cactorum* and *P. plurivora* isolates on hybrid poplar plants were performed on 20th June 2017, i.e., at day 0, using the underbark inoculation test at breast height. The plant stem diameter at breast height was 8.25 ± 0.21 (S.E.) cm. Both of the isolates used, *P. cactorum* isolate SFB057 (GenBank accession number JX276094) and *P. plurivora* isolate SFB182 (GenBank accession number KF234740), were isolated from mature poplar trees without visible crown symptoms that were surrounded by diseased trees. These isolates, previously found to be very aggressive to young poplar plants [14], were obtained from both soil and root samples using the baiting method. Control plants received mock treatments with sterile V8 agar plugs. Inoculated plants were regularly inspected and control re-isolations from randomly selected plants within both *Phytophthora* treatments were performed. Both gas exchange and chlorophyll *a* fluorescence measurements were conducted at day −1 (19/06) prior to infection to ensure there were no differences among the examined treatments. Subsequently, measurements were performed at days 3 (23/06), 10 (30/06), 20 (10/07), 30 (20/07), 38 (28/07), 43 (02/08), 52 (11/08), 66 (25/08), 79 (07/09), and 99 (27/09) post-inoculation (p.-i.). Measurements of VOCs were performed at days 9 (29/06) and 98 (26/09) p.-i., respectively. All measurements were conducted on seven randomly chosen plants per treatment.

### 2.2. Scanning Electron Microscopy

Bark and wood sections (transverse and radial surfaces) were cut using the sledge microtome (Reichert, Vienna, Austria), mounted on specimen stubs, sputter-coated with gold in the Sputter Coater K650X (Quorum Technologies, Ashford, UK) under argon atmosphere, and examined by high-vacuum scanning electron microscopy using a JEOL JSM-6390LV instrument (JEOL, Tokyo, Japan) operating at 20 kV.

### 2.3. Gas Exchange

An open portable photosynthesis system with infrared gas analyser LI-6400 XT (LI-COR, Lincoln, NE, USA) was used for in situ gas exchange measurements. Net photosynthetic rate (*P*_N_), transpiration (*E*), stomatal conductance (*g*_s_), and internal-to-ambient CO_2_ concentration ratio (*C*_i_/*C*_a_) were measured under a saturating photosynthetic photon flux density of 1500 ± 1 µmol m^−2^ s^−1^ and an ambient CO_2_ concentration of 400 ± 1 μmol mol^−1^ using the 6400-08 standard leaf chamber with the 6400-02B red/blue LED light source (LI-COR). Instantaneous water-use efficiency (WUE_inst_) was calculated as the ratio of *P*_N_ to *E* [36]. During measurements, microclimatic conditions inside the assimilation chamber were kept constant (air temperature 21 ± 1 °C, relative air humidity 65 ± 7%). The vapour pressure deficit was kept below 1.5 kPa. Measurements were performed on 3–4 leaves per plant.

### 2.4. Chlorophyll a Fluorescence

Chlorophyll *a* fluorescence yields were measured using a portable fluorometer Plant Efficiency Analyser (Hansatech Instruments Ltd., Kings Lynn, UK) as described by Ďurkovič et al. [37]. The parameters such as maximum photochemical efficiency of photosystem II (*F*_v_/*F*_m_), variable-to-initial fluorescence ratio (*F*_v_/*F*_0_), and potential electron acceptor capacity of photosystem II—“area” (i.e., area above the induction curve between *F*_0_ and *F*_m_) were determined. Measurements were performed on adaxial surfaces of 4–5 leaves per plant.

### 2.5. VOCs Measurements

Branches with intact leaves were sampled at the height of circa 3 m and immediately frozen at −80 °C. Then, 5–7 frozen leaves were cut into small pieces (0.5 g) and placed into 20 mL headspace (HS) vials that were sealed with an aluminium crimp cap with polytetrafluoroethylene/silicone septum. Vials with leaf pieces were heated at 90 °C for 20 min in an Agilent 7697A Headspace autosampler (Agilent Technologies, Santa Clara, CA, USA). The released VOCs were analysed by gas chromatography–mass spectrometry (GC–MS) using an Agilent 7890 A GC system connected to an Agilent 5975 C MSD system (Agilent Technologies). HS conditions were set as follows: helium was used as a carrier gas with a pressure 7.5 psi, oven temperature was set at 90 °C, vial equilibration time was 20 min, temperature at loop was 100 °C, and temperature of transfer line from HS to GC was 110 °C. GC–MS was conducted on a HP-5ms column with dimensions 30 m × 0.250 mm × 0.25 μm (Agilent Technologies), using helium as a carrier gas with a constant flow rate 1.0 mL min^−1^, at the temperature regime from 40 to 270 °C, and the injector temperature of 150 °C. Split conditions were set at 10:1, and the temperature of transfer line to MSD was 280 °C [38]. Two analytical replicates per plant were run. Volatiles were identified by comparison of their mass spectra with the NIST17 mass spectra library and the retention times of standards from the reference material Terpene Mixture 1 (Dr. Ehrenstorfer GmbH, Augsburg, Germany).

### 2.6. Statistical Analyses

Data were analysed by nested analysis of variance (plants were nested in *Phytophthora* treatments) using SAS/STAT 9.1 software (SAS Institute, Cary, NC, USA). The *Phytophthora* treatments were considered a fixed effect factor, whereas the plants were considered a random effect factor. In addition, the Euclidean distances were computed to identify major differences among the *Phytophthora* treatment observations during the growing season. First, both gas exchange and chlorophyll *a* fluorescence data were normalised, and then the Euclidean distances were computed by applying the scale and dist functions, respectively, using the R 4.0.3 software. Also, multivariate associations between independent VOC measurements were analysed using principal component analysis (PCA) to reveal a separation among the examined plants.

## 3. Results

### 3.1. Development of Pathogenic Symptoms

**Figure 1:**
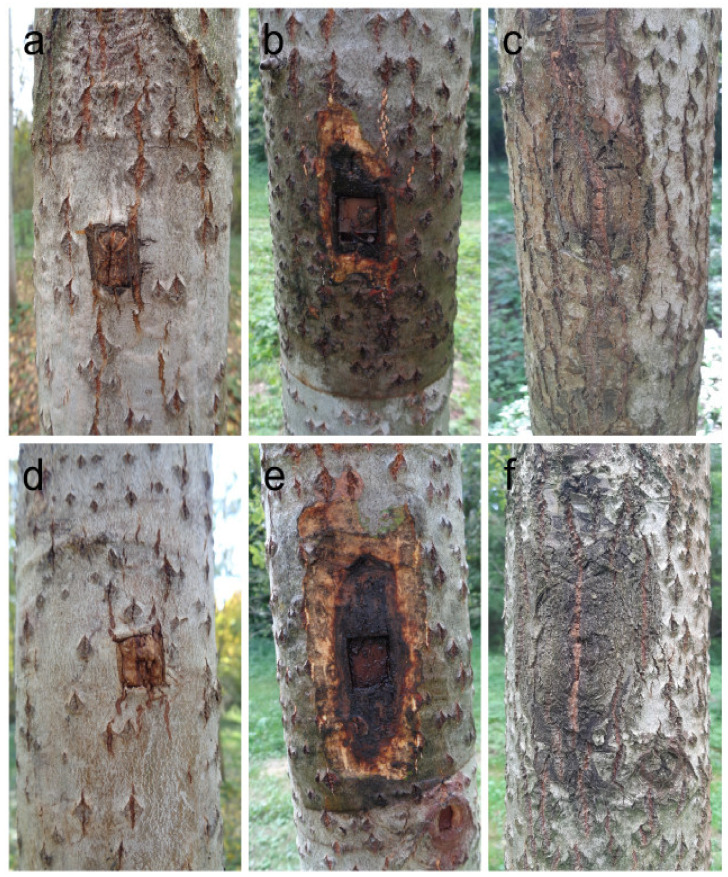
Symptoms caused by the *Phytophthora cactorum* isolate SFB057 (**b**,**c**) and the *Phytophthora plurivora* isolate SFB182 (**e**,**f**) on hybrid poplar stems following underbark inoculation test at breast height. (**a**,**d**) Controls showing the callus tissue which completely covered the wound sites. (**b**,**e**) Open bark cankers with necrotised bark tissues around the inoculation sites. (**c**,**f**) Bark cankers closed by the callus tissue four years following inoculations.

Following inoculation, *P. cactorum* induced a canker formation with the necrotic bark tissues having on average an axial length of 11.1 ± 1.9 (S.E.) cm, a tangential width of 5.9 ± 0.7 cm, and an area of 54.9 ± 12.8 cm^2^. Cankers induced by *P. plurivora* were not significantly greater having necrotic tissues with an average length of 14.3 ± 1.5 cm, width of 6.2 ± 1.0 cm, and area of 77.2 ± 18.1 cm^2^. The control plants showed no visible cankers or necroses and the callus tissue completely covered the wound sites (Figure 1a–f). Scanning electron microscopy revealed that the *Phytophthora* hyphae of both species, for the most part, occupied bark tissues, penetrating only very rarely to vessels or even to libriform fibres of the outermost annual growth ring (Figure S1a–f) and producing a dark coloration of the infected woody tissue zones. In response to the inoculations, there was no distinct formation of tyloses found in the vessels of host plants. Interestingly, only very scarce radial spread of hyphae through vessel pitting was observed, indicating that the two examined *Phytophthora* species prefer not to colonise lignified woody tissues in host woody plants.

### 3.2. Changes in Gas Exchange

**Figure 2:**
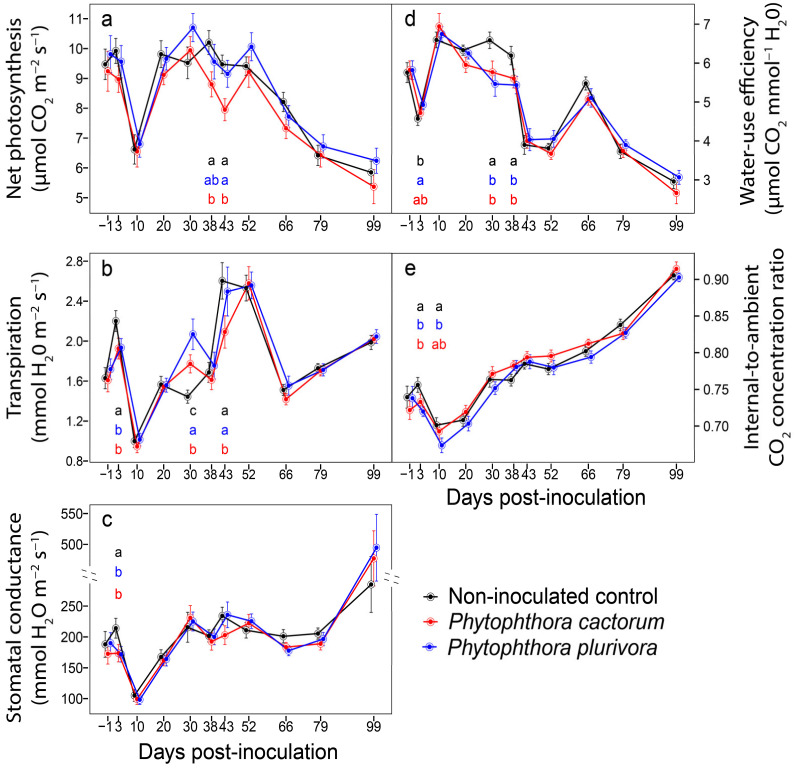
Gas exchange in hybrid poplar plants inoculated with *Phytophthora cactorum*, *Phytophthora plurivora*, and non-inoculated control plants. (**a**) Seasonal results of net CO_2_ assimilation rate. (**b**) Seasonal results of transpiration rate. (**c**) Seasonal results of stomatal conductance. (**d**) Seasonal results of instantaneous water-use efficiency. (**e**) Seasonal results of internal-to-ambient CO_2_ concentration ratio. Data represent means ± SE. Different letters indicate significant differences among the examined *Phytophthora* treatments (*p* ≤ 0.05). No letters indicate that there were no significant differences among the treatments (*p* > 0.05).

The results of gas exchange during the 99 day period of measurements are given in Figure 2. The plants inoculated with *P. cactorum* showed significantly reduced *P*_N_ rates compared to control plants at days 38 and 43 p.-i., whereas the plants inoculated with *P. plurivora* did not differ at all from control plants during the entire period of measurements. A superior photosynthetic performance by the plants inoculated with *P. plurivora* over those inoculated with *P. cactorum* was only found at day 43 p.-i.; otherwise, no differences between these two *Phytophthora* treatments were recorded (Figure 2a). With regard to rates of *E*, at day 3 p.-i., plants inoculated with either of the *Phytophthora* species responded by a drop in this trait. However, at day 30 p.-i., the plants having either of the *Phytophthora* treatments transpired a greater amount of water than the control plants, and the rate of *E* in the *P. plurivora* treatment was higher than that in the *P. cactorum* treatment. At day 43 p.-i. again, the plants inoculated with *P. cactorum* showed a reduced rate of *E* compared to both the plants inoculated with *P. plurivora* and the control plants. After day 43 p.-i., the differences in *E* among *Phytophthora* treatments and control plants were negligible (Figure 2b). The only difference in *g*_s_ was found at day 3 p.-i. when all the plants treated with either one of the two *Phytophthora* treatments showed lower values compared to the control plants. During the remaining period of measurements, no differences among *Phytophthora* treatments and control plants were observed (Figure 2c). With regard to the instantaneous photosynthetic carbon gain per unit transpirational water vapor loss, at day 3 p.-i., the plants inoculated with *P. plurivora* achieved higher values than the control plants. On the other hand, the performance of the control plants was superior to either one of the two *Phytophthora* treatments for WUE_inst_ at days 30 and 38 p.-i. Following this, no differences were found among *Phytophthora* treatments and control plants (Figure 2d). The differences in instantaneous *C*_i_/*C*_a_ were observed only at days 3 and 10 p.-i. At day 3 p.-i., control plants achieved a higher ratio (due to a lower diffusion resistance of stomata) than either of the two *Phytophthora* treatments, whereas at day 10 p.-i., the control plants showed a higher value only over the plants inoculated with *P. plurivora*. During the remaining period of measurements, there were no differences observed among the *Phytophthora* treatments and the control plants (Figure 2e).

During measurements of *P*_N_, *E*, *g*_s_, and *C*_i_/*C*_a_, a rapid drop in values for these parameters was found at day 10 p.-i. (for WUE_inst_ it was the opposite effect, i.e., a rapid increase). This drop was typical not only for each of the *Phytophthora* treatments but also for the control plants. An explanation for this observation stems from the microclimatic conditions in the experimental field plot during the preceding weeks when dry and hot weather conditions were accompanied by a drop in soil water potential (Figure S2). These conditions indicate a long-lasting and limited availability of water which resulted in a negative effect on gas exchange parameters for all treatments and control plants.

### 3.3. Changes in Chlorophyll a Fluorescence

**Figure 3:**
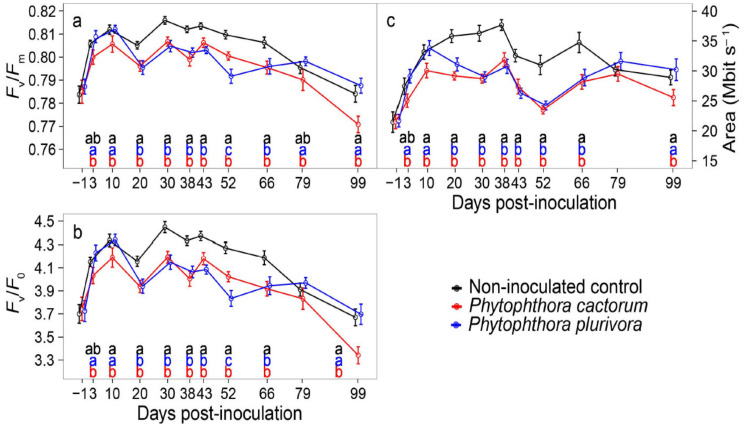
Chlorophyll *a* fluorescence yields in hybrid poplar plants inoculated with *Phytophthora cactorum*, *Phytophthora plurivora*, and non-inoculated control plants. (**a**) Seasonal results of maximum photochemical efficiency of photosystem II. (**b**) Seasonal results of variable-to-initial fluorescence ratio. (**c**) Seasonal results of potential electron acceptor capacity of photosystem II. Data represent means ± SE. Different letters indicate significant differences among the examined *Phytophthora* treatments (*p* ≤ 0.05). No letters indicate that there were no significant differences among the treatments (*p* > 0.05).

For both *F*_v_/*F*_m_ and *F*_v_/*F*_0_ ratios, the inoculated plants responded quite similarly (Figure 3). Plants inoculated with *P. cactorum* achieved lower chlorophyll *a* fluorescence yields than the control plants from day 10 p.-i. to the end of the measurement period at day 99 p.-i., with the only exception being at day 79 p.-i. On that day, both *F*_v_/*F*_m_ and *F*_v_/*F*_0_ ratios were similar to those in the control plants. Plants inoculated with *P. plurivora* had lower yields than the control plants for a shorter period, i.e., from day 20 p.-i. to day 66 p.-i. Interestingly, day 52 p.-i. was the only day of measurements when the *P. cactorum* treatment achieved higher *F*_v_/*F*_m_ and *F*_v_/*F*_0_ ratios than the *P. plurivora* treatment (Figure 3a,b). With regard to the parameter “area”, the course of measurements resembled much the same as the *F*_v_/*F*_0_ ratio, with the only exception being at day 52 p.-i. when the differences between the two *Phytophthora* treatments were negligible (Figure 3c).

In addition, multivariate gas exchange and chlorophyll *a* fluorescence analysis based on the Euclidean distances (Table 1) revealed the particular days p.-i. that show the greatest separation among the examined treatments. Plants inoculated with *P. cactorum* were separated the most from the control plants at days 30 and 38 p.-i., whereas the greatest separation of plants inoculated with *P. plurivora* from the control plants occurred at day 30 p.-i. and also belatedly at day 52 p.-i. Thus, these particular days p.-i. may indicate the period with the most stressful and harmful effects of *Phytophthora* hyphae on the physiological performance and/or host responses of inoculated plants. Interestingly, the greatest separation between the two *Phytophthora* treatments was identified at day 99 p.-i. when accounting primarily for the significant differences in chlorophyll *a* fluorescence yields.

### 3.4. Changes in VOCs

At day 9 p.-i., 23 VOCs were identified in the control plants and both *Phytophthora* treatments. Three VOCs were present in more than the 10% relative proportion, namely 2-hexenal, 2-ethylfuran and hexanal, whereas the remaining 20 compounds were present in lesser amounts. The *P. cactorum* treatment contained six compounds in a higher abundance than both the control plants and the *P. plurivora* treatment. The *P. plurivora* treatment contained 12 compounds in a higher abundance than both the control plants and the *P. cactorum* treatment. The control plants contained five compounds that were more abundant than either *Phytophthora* treatment. In addition, ocimene was found to be a season-specific compound that was not emitted later in the season at day 98 p.-i. (Table 2).

At day 98 p.-i., 32 VOCs were identified in all treatments. Twenty-two compounds were emitted constitutively, whereas 10 season-specific compounds (phenylethyl alcohol, α-cubebene, 2-undecenal, copaene, aloaromadendrene, germacrene D, α-muurolene, β-muurolene, γ-muurolene, and naphthalene) were found only at the end of the growing season, not at the early day 9 p.-i. in June. Most importantly, the emissions of both α-cubebene and germacrene D were induced by the two *Phytophthora* treatments, whereas these compounds were not found in the control plants. Three compounds, i.e., 2-ethylfuran, 3-hexen-1-ol, and 2-hexenal, showed higher than the 10% relative proportion. The *P. cactorum* treatment contained two compounds, with one of them germacrene D, in a higher abundance than both the control plants and the *P. plurivora* treatment. The *P. plurivora* treatment contained 24 compounds, with one of them α-cubebene, in a higher abundance than both the control plants and the *P. cactorum* treatment. Finally, the control plants contained 6 compounds that were more abundant than either *Phytophthora* treatment (Table 3).

**Figure 4:**
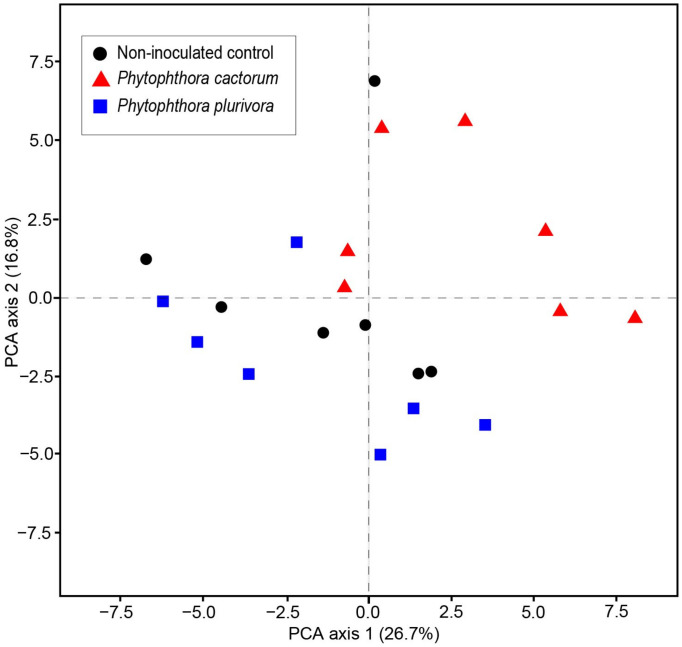
Positions of the examined plants on the first and second axes of the principal component analysis (PCA) based on the measurements of volatile organic compounds at days 9 and 98 p.-i. Control plants, plants inoculated with *Phytophthora cactorum* and plants inoculated with *Phytophthora plurivora* are as indicated in the key.

In addition, PCA based on multivariate associations among the identified VOCs showed that the two *Phytophthora* species were oppositely separated from the control plants (Figure 4). The majority of the plants inoculated with *P. cactorum* were distributed through the positive side of the first PCA axis and, at the same time, through the positive side of the second PCA axis. On the other hand, plants inoculated with *P. plurivora* were distributed mostly through the negative side of the second PCA axis.

### 3.5. Correlations between Bark Canker Size and Leaf VOCs

**Figure 5:**
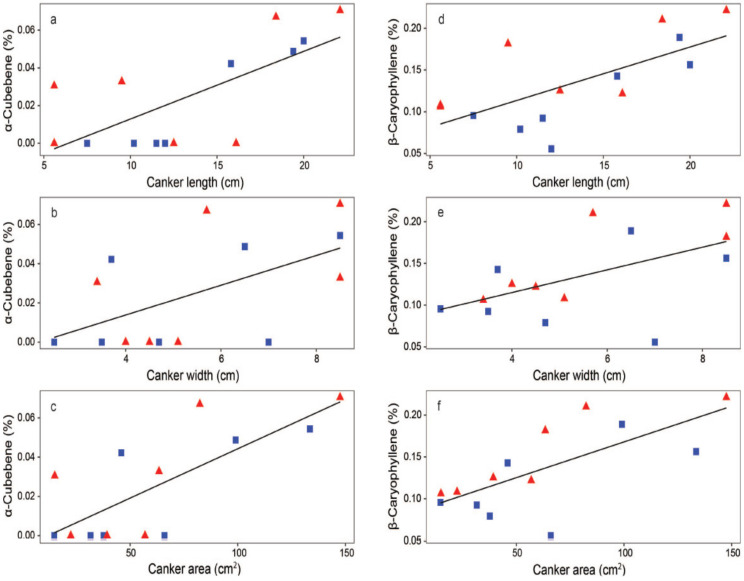
Correlations between bark canker size and leaf volatile sesquiterpenes identified in the hybrid poplar plants. (**a**) Relationship of canker length to the emission of α-cubebene. (**b**) Relationship of canker width to the emission of α-cubebene. (**c**) Relationship of canker area to the emission of α-cubebene. (**d**) Relationship of canker length to the emission of β-caryophyllene. (**e**) Relationship of canker width to the emission of β-caryophyllene. (**f**) Relationship of canker area to the emission of β-caryophyllene. Red triangles show the plants inoculated with *Phytophthora cactorum*; blue squares show the plants inoculated with *Phytophthora plurivora*.

Significant relationships were found between the size of the bark cankers and several VOCs. Axial length, tangential width, and the area of cankers positively correlated with the relative proportions of both α-cubebene and β-caryophyllene in the volatile blend. The greater the canker length (*r* = 0.70, *p* = 0.005, Figure 5a), canker width (*r* = 0.56, *p* = 0.036, Figure 5b), and canker area (*r* = 0.75, *p* = 0.002, Figure 5c), the greater the relative proportion of α-cubebene was for the plants emitting this sesquiterpene. Similarly, the greater the canker length (*r* = 0.68, *p* = 0.007, Figure 5d), width (*r* = 0.55, *p* = 0.041, Figure 5e), and area (*r* = 0.70, *p* = 0.005, Figure 5f), the greater the emissions of β-caryophyllene were. In addition, we found other significant correlations. The greater the canker length and canker area, the greater the emissions for copaene, aloaromadendrene, cadinene, naphthalene, and α-muurolene, β-muurolene and γ-muurolene isomers were. With regard to germacrene D, we found that the greater axial length of the canker triggered a greater emission of this sesquiterpene (*r* = 0.54, *p* = 0.046). These results reveal very interesting findings that, while all the above VOCs responded sensitively to the axial development of bark cankers, only α-cubebene and β-caryophyllene were emitted in increased amounts following the tangential spread of the cankers.

## 4. Discussion

In this study, stem inoculation pathogenicity tests were performed on 10-year-old hybrid poplar plants growing under field conditions when using two known generalist pathogens of the genus *Phytophthora*. After initial well development of the pathogens within the tissue, typical cankers and lesions were observed together with a slight flow of dark exudates. The two *Phytophthora* species were easily isolated during control re-isolations. However, for long-lived forest trees, a 99 day-long experiment is not a sufficiently long period to judge whether or not the inoculated plants of the hybrid poplar are tolerant to the disease. When inspecting the plants four years following inoculations, it was found that in the majority of the inoculated plants, the callus tissue had already closed over the bark cankers, and neither of the *Phytophthora* species colonizing the bark tissues could be reisolated. All inoculated plants (for both *P. cactorum* and *P. plurivora* treatments) continue to grow vigorously without any tendency towards leaf wilting, yellowing, or premature defoliation, and no hybrid poplar plant, to date, has died under field conditions. This result contrasts the previously obtained results under laboratory conditions, where both *P. cactorum* and *P. plurivora* were found to be very aggressive towards the young poplar plants of two different clones [14]. In addition, in the underbark pathogenicity test performed on 1 year-old saplings of *Populus alba*, 33% of plants were girdled, whereas the majority of the tested saplings had closed wounds with the callus tissue and survived the infection [15].

Several studies reported that the results of pathogenicity tests in glasshouse experiments correlate with the results achieved under field conditions. This suggests the former approach to be a useful, handy, and cheaper tool for the aggressiveness evaluation, particularly in screening resistance programmes for various agricultural crops [39,40]. In the case of some forest pathogens, a similar situation was recorded when testing the susceptibility of young *Pinus sylvestris* of similar ages under both conditions [41] or during tests on young *Quercus petraea* under glasshouse conditions and mature *Q. petraea* under field conditions [42]. However, the opposite results were also recorded when these two experimental approaches were compared. For example, a pathogenic fungus *Neonectria punicea* was aggressive towards juvenile *Fraxinus excelsior* under field conditions [43], while it produced no symptoms in young plants when the pathogenicity test was performed under laboratory conditions (Milenković, unpublished). Although less frequently performed and having several disadvantages, pathogenicity tests on larger trees under field conditions show a more realistic scenario for the particular pathogens. Based upon these and previously mentioned contrasting results on poplar plants, improved methods and assays for testing the susceptibility of various host plants are required.

Photosynthesis is one of the most sensitive physiological indicators of stress caused by different abiotic and biotic factors. A change in PSII efficiency and overall assimilation rate due to root damage by *Phytophthora* pathogens has been previously reported [44,45]. Root damage leads directly to a drop in water supply, water potential, and to a hinderance of all metabolic processes. Colonisation of even a tiny part of the root system may quickly lead to a hormonal imbalance, especially influencing abscisic acid responsible for stomatal closure [46]. On the other hand, trunk damage may result in xylem dysfunction and eventually death due to constrained water and mineral transport, or the *Phytophthora* may destroy cortex and phloem cells, leading to a slower progression of visible symptoms. Even without direct destruction of the root system, fine root growth is slowed considerably when efficient phloem transport is heavily impaired. This causes starch accumulation in the leaves and the consequent closure of stomata reducing CO_2_ assimilation, even when water availability is not restricted [47]. We did not confirm such stomata closure during the experiment, presumably due to the mild damage of phloem tissue and minimal damage to xylem.

We confirmed significant differences among the treatments in some gas exchange traits, mainly during the period with limited water availability (i.e., at days 30–43 p.-i.). The CO_2_ assimilation rate was lower in plants inoculated with *P. cactorum*, and water-use efficiency was highest in non-inoculated plants, suggesting a slight restriction of metabolic processes caused by the disease. However, these differences were not observed later during the experiment. The differences in transpiration rate among treatments were inconsistent during the growing season and minimal differences in stomatal conductance were observed among both of the *Phytophthora* treatments and the control. Therefore, we assume that alteration in phloem transport rather than xylem transport was a restrictive factor. The majority of studies dealing with the controlled artificial inoculation focus just on seedlings and young plants. Clearly, the susceptibility of the younger plants is much greater than that of the mature plants [48]. Dinis et al. [49] revealed a significant decrease in the CO_2_ assimilation rate for a sensitive variety of *Castanea sativa* nine days p.-i. with *P. cinnamomi*, while the values for a resistant variety of chestnut did not decrease or were even higher. In that study, the authors examined three-year-old seedlings. Clemenz et al. [47] also observed a decrease in CO_2_ uptake and transpiration rate within the growing season for three-year-old individuals of *Alnus glutinosa* inoculated with *P*. × *alni* (Brasier and S.A. Kirk) Husson, Ioos, and Marçais. Similar to our results, Umami et al. [50] confirmed no drought-like symptoms due to an infection with *P. cinnamomi* in two-year-old *Eucalyptus obliqua* within 28 days p.-i. The authors hypothesised that the reason could be a delayed response, lower aggressivity of the used strain, or a higher plant resistance. We found no substantial changes in gas exchange traits within the 99 day-long experiment in infected plants compared to the control.

Dinis et al. [49] reported nonsignificant changes in the maximum photochemical efficiency of photosystem II (*F*_v_/*F*_m_) of *Castanea sativa* inoculated with *P. cinnamomi* in an 18 day ongoing experiment. However, there is a higher risk of reactive oxygen species being formed during the infection, affecting the xanthophyll cycle [51]. This might have influenced the light-dependent stage of photosynthesis in our experiment performed under field conditions: the differences in chlorophyll *a* fluorescence were not huge but were significant and more consistent compared to gas exchange. Both *Phytophthora* treatments showed lower efficiency and potential electron acceptor capacity of photosystem II during the summer period. The plants inoculated with *P. cactorum* were more affected at the end of the growing season compared to non-inoculated plants. The plants inoculated with *P. plurivora* regenerated and values measured during September were similar to those measured on healthy control plants. This fact points to the more harmful effect of *P. cactorum* on physiological processes compared to *P. plurivora*.

VOCs released from the infected plant tissues invoke mechanisms that protect the plant against biotic and abiotic stresses. In this study, the highest constitutive emissions of VOCs were found for the aldehydes 2-hexenal and hexanal as well as for the heterocyclic compound 2-ethylfuran (9 days p.-i.), and again for 2-ethylfuran, 2-hexenal, and the alcohol 3-hexen-1-ol (98 days p.-i.), respectively. In addition, the largest differences following *Phytophthora* inoculations were observed mostly among the major components of the volatile blend. Interestingly, Bate and Rothstein [52] reported that (*E*)-2-hexenal induced a subset of genes acting in the phenylpropanoid-related and lipoxygenase pathways that are involved in defence responses of *Arabidopsis thaliana*. Previous studies on VOC emissions in poplars reported negligible constitutive levels of monoterpenes and sesquiterpenes [53,54]. In agreement with these findings, we observed that, among the monoterpenes and sesquiterpenes, only 3-carene (9 days p.-i.) and cadinene (98 days p.-i.) were constitutively emitted in amounts higher than 1%. However, the induction of specific VOC emissions may lead to a direct resistance to pathogens in the emitter itself [55,56]. In this study, we identified two sesquiterpenes, α-cubebene and germacrene D, the emissions of which were induced solely by the *Phytophthora* treatments. Chen et al. [57] reported that *Bacillus* isolates colonizing *Vanilla planifolia* beans were capable of glucovanillin hydrolysis. During conversion, the major VOCs emitted included vanillin, α-cubebene, β-pinene, and guaiacol. Recent findings concerning α-iso-cubebene isolated from *Schisandra chinensis* fruit revealed that this natural compound shows anti-inflammatory, antioxidative, and immunomodulatory activities, in addition to ameliorating the symptoms of cardiovascular disease [58,59]. More interestingly, α-iso-cubebene can reverse the progression of toxic shock by triggering multiple protective downstream signalling pathways to enhance the elimination of invading pathogens [60]. The biosynthetic pathway leading to the formation of the isomeric compounds α-cubebene and β-cubebene via (*S*)-(–)-germacrene D as intermediate was reported recently in *Vitis vinifera* [61]. In many plants, germacrenes play a central role as important intermediates in the biosynthesis of different sesquiterpene derivatives [62,63]. Moreover, germacrenes B and D both show antimicrobial and insecticidal properties [64,65]. In *Zea mays* seedlings attacked by lepidopteran larvae *Spodoptera littoralis*, germacrene D was present in a complex mixture of herbivore-induced plant volatiles that attracted females of the parasitic wasp *Cotesia marginiventris* with a previous oviposition experience in the lepidopteran larvae [66]. However, the compound itself was not solely responsible for attracting a natural enemy. On the other hand, both α-cubebene and β-cubebene isomers as well as germacrene D were found to be the major volatile compounds that were able to distinguish different blends of gypsy moth (*Lymantria dispar*)-damaged and undamaged foliage in *Populus nigra* [67]. Except for the emission of β-cubebene, these observations support our findings in the hybrid poplar inoculated with two *Phytophthora* species. From the standpoint of defence responses, both α-cubebene and germacrene D are candidates that may function as signal molecules for the suppression of *Phytophthora* hyphae spread from necrotic sites of the bark to healthy living tissues. The effects of α-cubebene and germacrene D-induced emissions were also strengthened by the biological activities of β-caryophyllene that was previously shown to exhibit, among others, strong antioxidative, antimicrobial, and antifungal properties [68,69,70].

## Figures and Tables

**Table 1:** The Euclidean distances showing differences among the examined treatments during the course of gas exchange and chlorophyll *a* measurements. Numbers given in bold indicate days post-inoculation in which the treatments were the most separated.

Day Post-Inoculation	Control—*P. cactorum*	Control—*P. plurivora*	*P. cactorum*—*P. plurivora*
−1	0.4322	0.4721	0.6032
3	1.4142	1.1865	1.5981
10	1.1818	0.5246	1.3273
20	2.1093	1.7172	0.6922
30	**2.5246**	**3.0534**	0.9813
38	**2.5568**	2.3644	0.7698
43	2.2664	2.1817	1.3858
52	2.2846	**3.0032**	1.347
66	2.3166	2.0586	0.5557
79	0.6835	0.576	1.1057
99	2.1029	0.8082	**2.5451**

**Table 2:** Leaf volatile organic compounds (VOCs) identified in hybrid poplar at day 9 post-inoculation. VOC abundance values are expressed as a ratio to the maximum value for the particular compound across each row. RT, retention time.

RT (min)	Compound	VOC Frequency in Plants			VOC Abundance (Relative Proportion in %)		
		Control	*P*. *cactorum*	*P*. *plurivora*	Control	*P*. *cactorum*	*P*. *plurivora*
3.114	Furan, 2-ethyl-	7/7	7/7	7/7	0.731 (22.538)	0.667 (27.729)	1.000 (26.564)
4.740	Hexanal	7/7	7/7	7/7	0.915 (17.452)	0.494 (12.708)	1.000 (16.438)
5.962	2-Hexenal	7/7	7/7	7/7	0.923 (22.854)	0.533 (17.817)	1.000 (21.336)
6.057	3-Hexen-1-ol, (*Z*)-	7/7	7/7	7/7	1.000 (7.193)	0.841 (8.160)	0.969 (6.005)
6.367	2-Hexen-1-ol, (*Z*)-	7/7	7/7	7/7	0.963 (1.681)	1.000 (2.355)	0.322 (0.485)
7.245	Heptanal	7/7	7/7	7/7	0.847 (0.237)	0.570 (0.215)	1.000 (0.241)
7.447	2,4-Hexadienal, (*E*,*E*)-	7/7	7/7	7/7	0.967 (0.727)	0.790 (0.801)	1.000 (0.648)
8.878	Benzaldehyde	7/7	7/7	7/7	0.696 (0.454)	1.000 (0.879)	0.624 (0.350)
9.823	Furan, 2-pentyl-	7/7	7/7	7/7	0.983 (0.239)	0.915 (0.299)	1.000 (0.209)
10.107	2-(2-Pentenyl)furan	7/7	7/7	7/7	0.948 (0.422)	1.000 (0.600)	0.964 (0.370)
10.377	2,4-Heptadienal, (*E*,*E*)-	7/7	7/7	7/7	0.967 (0.117)	0.745 (0.121)	1.000 (0.104)
11.322	Benzaldehyde, 2-hydroxy-	7/7	7/7	7/7	0.655 (0.228)	1.000 (0.469)	0.578 (0.173)
11.511	Ocimene	7/7	7/7	7/7	0.782 (0.061)	0.872 (0.091)	1.000 (0.067)
13.158	Nonanal	7/7	7/7	7/7	0.965 (0.608)	0.699 (0.594)	1.000 (0.543)
15.804	Benzoic acid, 2-hydroxy-, methyl ester	7/7	7/7	7/7	0.856 (0.193)	1.000 (0.304)	0.639 (0.124)
16.100	Decanal	7/7	7/7	7/7	1.000 (0.064)	0.877 (0.076)	0.993 (0.055)
16.573	1-Cyclohexene-1-carboxaldehyde, 2,6,6-trimethyl-	7/7	7/7	7/7	0.958 (0.096)	0.842 (0.114)	1.000 (0.086)
17.113	1,3,8-*p*-Menthatriene	3/7	7/7	7/7	0.717 (0.042)	1.000 (0.078)	0.797 (0.040)
17.464	3-Carene	7/7	7/7	7/7	0.658 (1.320)	0.356 (0.962)	1.000 (1.729)
20.272	Eugenol	7/7	7/7	7/7	1.000 (0.071)	0.754 (0.072)	0.825 (0.050)
21.960	β-Caryophyllene	7/7	7/7	7/7	0.810 (0.051)	0.784 (0.066)	1.000 (0.054)
23.566	β-Ionone	7/7	7/7	7/7	1.000 (0.060)	0.817 (0.066)	0.877 (0.045)
24.498	Cadinene	5/7	6/7	4/7	1.000 (0.027)	0.938 (0.034)	0.749 (0.017)

**Table 3:** Leaf volatile organic compounds (VOCs) identified in hybrid poplar at day 98 post-inoculation. VOC abundance values are expressed as a ratio to the maximum value for the particular compound across each row. RT, retention time.

RT (min)	Compound	VOC Frequency in Plants			VOC Abundance (Relative Proportion in %)		
		Control	*P. cactorum*	*P. plurivora*	Control	*P. cactorum*	*P. plurivora*
3.114	Furan, 2-ethyl-	7/7	7/7	7/7	1.000 (21.294)	0.770 (23.863)	0.912 (16.349)
4.74	Hexanal	7/7	7/7	7/7	0.985 (5.731)	0.488 (4.134)	1.000 (4.900)
5.962	2-Hexenal	7/7	7/7	7/7	0.935 (13.699)	0.635 (13.547)	1.000 (12.341)
6.057	3-Hexen-1-ol, (*Z*)-	7/7	7/7	7/7	0.703 (18.660)	0.495 (19.100)	1.000 (22.348)
6.367	2-Hexen-1-ol, (*Z*)-	7/7	7/7	7/7	0.613 (4.118)	0.151 (1.475)	1.000 (5.653)
7.245	Heptanal	7/7	7/7	7/7	0.970 (0.258)	0.570 (0.221)	1.000 (0.224)
7.447	2,4-Hexadienal, (*E*,*E*)-	7/7	7/7	7/7	0.992 (0.607)	0.684 (0.609)	1.000 (0.515)
8.878	Benzaldehyde	7/7	7/7	7/7	0.836 (1.346)	0.456 (1.069)	1.000 (1.356)
9.823	Furan, 2-pentyl-	7/7	7/7	7/7	0.866 (0.631)	0.680 (0.720)	1.000 (0.613)
10.107	2-(2-Pentenyl)furan	7/7	7/7	7/7	0.985 (1.385)	0.867 (1.773)	1.000 (1.184)
10.377	2,4-Heptadienal, (*E*,*E*)-	7/7	7/7	7/7	1.000 (0.153)	0.764 (0.170)	0.847 (0.109)
11.322	Benzaldehyde, 2-hydroxy-	7/7	7/7	7/7	0.793 (2.187)	0.371 (1.487)	1.000 (2.322)
13.158	Nonanal	7/7	7/7	7/7	0.861 (0.536)	0.699 (0.633)	1.000 (0.524)
13.441	Phenylethyl Alcohol	1/7	4/7	7/7	0.068 (0.087)	0.039 (0.073)	1.000 (1.083)
15.804	Benzoic acid, 2-hydroxy-, methyl ester	7/7	7/7	7/7	1.000 (2.351)	0.616 (2.107)	0.952 (1.885)
16.1	Decanal	7/7	7/7	7/7	0.951 (0.115)	0.776 (0.136)	1.000 (0.102)
16.573	1-Cyclohexene-1-carboxaldehyde, 2,6,6-trimethyl-	7/7	7/7	7/7	1.000 (0.122)	0.774 (0.137)	0.903 (0.093)
17.113	1,3,8-*p*-Menthatriene	7/7	7/7	7/7	0.959 (0.247)	0.445 (0.167)	1.000 (0.217)
17.464	3-Carene	3/7	3/7	7/7	0.201 (0.242)	0.081 (0.142)	1.000 (1.011)
20.124	α-Cubebene	0/7	4/7	3/7	0.000 (0.000)	0.969 (0.032)	1.000 (0.019)
20.272	Eugenol	7/7	7/7	7/7	1.000 (0.262)	0.520 (0.198)	0.982 (0.217)
20.427	2-Undecenal	4/7	7/7	7/7	0.251 (0.151)	1.000 (0.875)	0.392 (0.198)
20.812	Copaene	7/7	7/7	7/7	0.795 (0.136)	0.811 (0.201)	1.000 (0.144)
21.96	β-Caryophyllene	7/7	7/7	7/7	0.857 (0.113)	0.813 (0.156)	1.000 (0.111)
22.999	Aloaromadendrene	7/7	7/7	7/7	0.861 (0.238)	0.865 (0.349)	1.000 (0.233)
23.364	β-Muurolene	7/7	7/7	7/7	0.813 (0.184)	0.827 (0.271)	1.000 (0.190)
23.499	Germacrene D	0/7	7/7	5/7	0.000 (0.000)	1.000 (0.083)	0.813 (0.039)
23.566	β-Ionone	7/7	6/7	6/7	1.000 (0.062)	0.536 (0.048)	0.847 (0.044)
23.944	α-Muurolene	7/7	7/7	7/7	0.882 (0.436)	0.855 (0.615)	1.000 (0.416)
24.282	γ-Muurolene	7/7	7/7	7/7	0.647 (0.471)	0.736 (0.780)	1.000 (0.613)
24.498	Cadinene	7/7	7/7	7/7	0.884 (1.088)	0.907 (1.623)	1.000 (1.036)
24.835	1,2,4a,5,6,8a-hexahydro-4,7-dimethyl-1-(1-methylethyl)-,[1R-(1.alpha.,4a.alpha.,8a.alpha.)]-naphthalene	7/7	7/7	7/7	0.865 (0.098)	0.858 (0.142)	1.000 (0.096)

## Data Availability

The data presented in this study are available on request from the corresponding author. The data are not publicly available due to no big datasets for deposition to public repositories.

## Supplementary Materials

The following are available online at https://www.mdpi.com/article/10.3390/jof7110969/s1, Figure S1: Scanning electron microscopy images of *Phytophthora cactorum* (A–C) and *Phytophthora plurivora* (D–F) hyphae. (A) Mycelium of *P. cactorum* colonizing bark tissues, cross-section, scale bar = 100 μm. (B) Hypha spreading through the libriform fibre, radial section, scale bar = 5 μm. (C) Hypha with a globose oogonium spreading through the vessel pitting area but avoiding penetration through the pit, radial section, scale bar = 2 μm. (D) Mycelium of *P. plurivora* colonizing bark tissues, cross-section, scale bar = 100 μm. (E) Hyphae spreading through both the vessel and the libriform fibre, radial section, scale bar = 20 μm. (F) Hypha spreading through the vessel pitting area but avoiding penetration through the pits, radial section, scale bar = 10 μm. Figure S2: The Walter and Lieth climate diagram for the experimental field plot for the year 2017 distinguishes dry (yellow) and humid (blue) periods. Temperature (red line) refers to the average monthly mean temperature, and precipitation (blue line) refers to the average monthly precipitation. Daily averages of hourly recorded soil water potential (SWP, black line) and precipitation (blue columns) are displayed in the bottom of the figure. Both annual average air temperature and precipitation total are as indicated in the key.
