# Supplementary material for: Effects of Phytophthora Inoculations on Photosynthetic Behaviour and Induced Defence Responses of Plant Volatiles in Field-Grown Hybrid Poplar Tolerant to Bark Canker Disease
Source: J Fungi (Basel). 2021 Nov 15;7(11):969. doi: 10.3390/jof7110969 (PMC8624009; doi:10.3390/jof7110969)
Supplement: Supplementary file 1 [file jof-07-00969-s001.zip › jof-1419398-supplementary.pdf]

Supplementary Figures

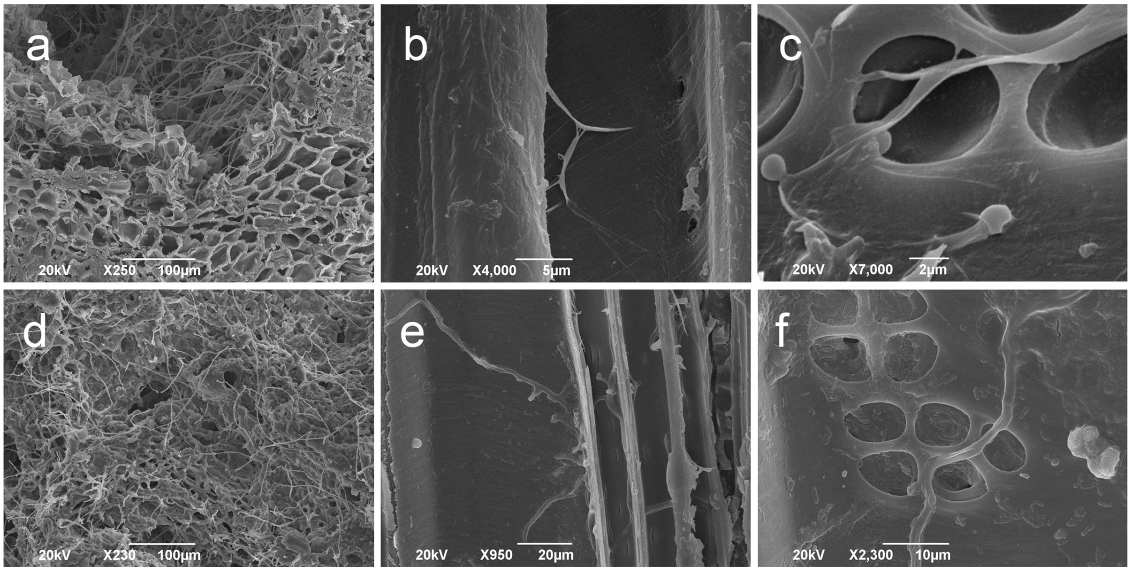

Supplementary Figure S1. Scanning electron microscopy images of *Phytophthora cactorum* (A–C) and *Phytophthora plurivora* (D–F) hyphae. (**A**) Mycelium of *P. cactorum* colonizing bark tissues, cross-section, scale bar = 100 μm. (**B**) Hypha spreading through the libriform fibre, radial section, scale bar = 5 μm. (**C**) Hypha with a globose oogonium spreading through the vessel pitting area but avoiding penetration through the pit, radial section, scale bar = 2 μm. (**D**) Mycelium of *P. plurivora* colonizing bark tissues, cross-section, scale bar = 100 μm. (**E**) Hyphae spreading through both the vessel and the libriform fibre, radial section, scale bar = 20 μm. (**F**) Hypha spreading through the vessel pitting area but avoiding penetration through the pits, radial section, scale bar = 10 μm.

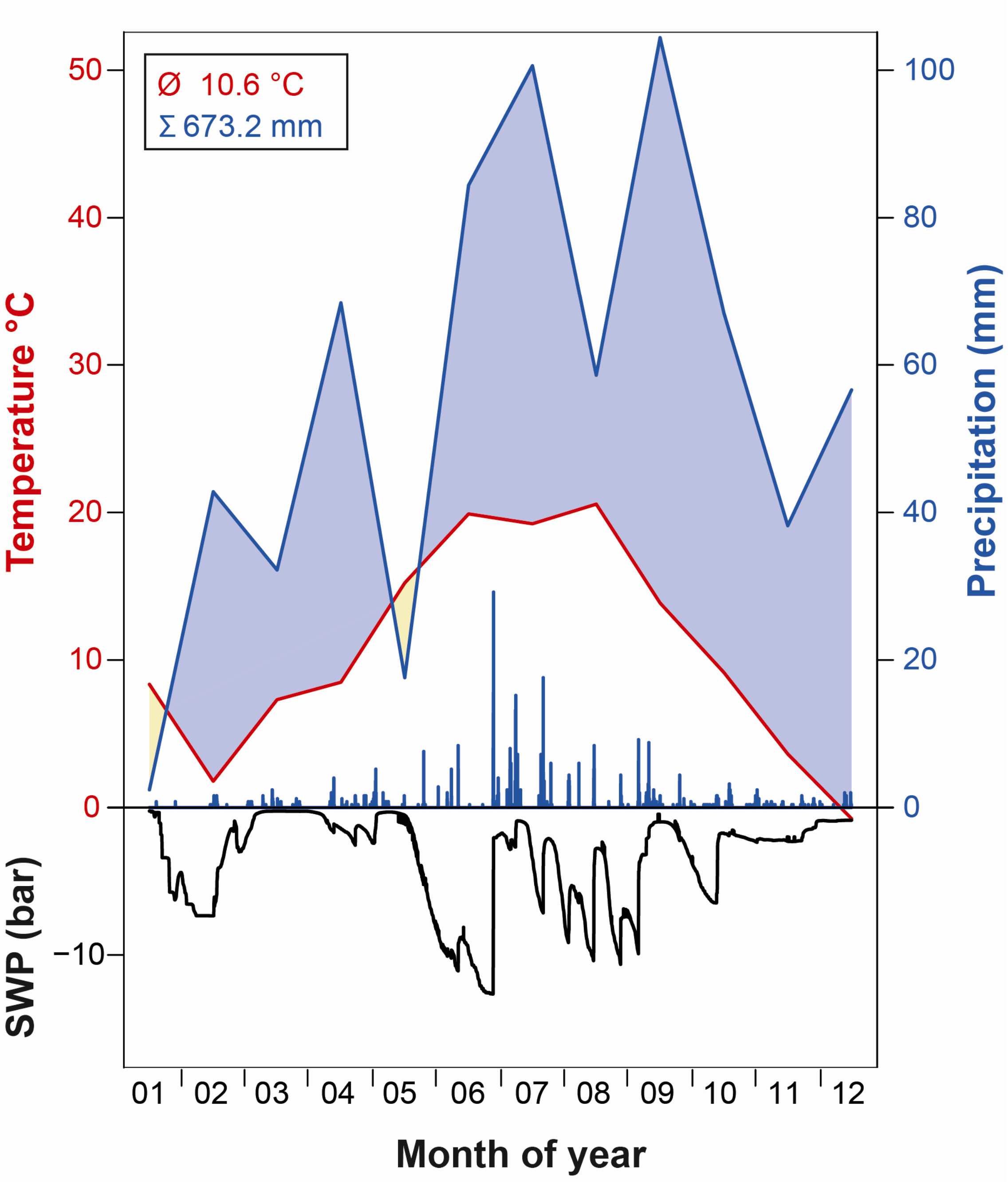

Supplementary Figure S2. The Walter and Lieth climate diagram for the experimental field plot for the year 2017 distinguishes dry (yellow) and humid (blue) periods. Temperature (red line) refers to the average monthly mean temperature, and precipitation (blue line) refers to the average monthly precipitation. Daily averages of hourly recorded soil water potential (SWP, black line) and precipitation (blue columns) are displayed in the bottom of the figure. Both annual average air temperature and precipitation total are as indicated in the key.
